# Supplementary material for: Untargeted Metabolomics on Skin Mucus Extract of Channa argus against Staphylococcus aureus: Antimicrobial Activity and Mechanism
Source: Foods. 2021 Dec 4;10(12):2995. doi: 10.3390/foods10122995 (PMC8701811; doi:10.3390/foods10122995)
Supplement: Supplementary file 1 [file foods-10-02995-s001.zip › foods-1455883-supplementary.pdf]

Table S1. PLS-DA cross-validation details

Measure	1 comps	2 comps	3 comps	4 comps	5 comps
Accuracy	1.0	1.0	1.0	1.0	1.0
R2	0.90656	0.96898	0.993	0.99824	0.99941
Q2	0.8572	0.94947	0.96161	0.96433	0.96826
